# Supplementary material for: Checkpoint kinase 1 is essential for normal B cell development and lymphomagenesis
Source: Nat Commun. 2017 Nov 22;8:1697. doi: 10.1038/s41467-017-01850-4 (PMC5700047; doi:10.1038/s41467-017-01850-4)
Supplement: Supplementary file 1 — Supplementary Information [file 41467_2017_1850_MOESM1_ESM.pdf]

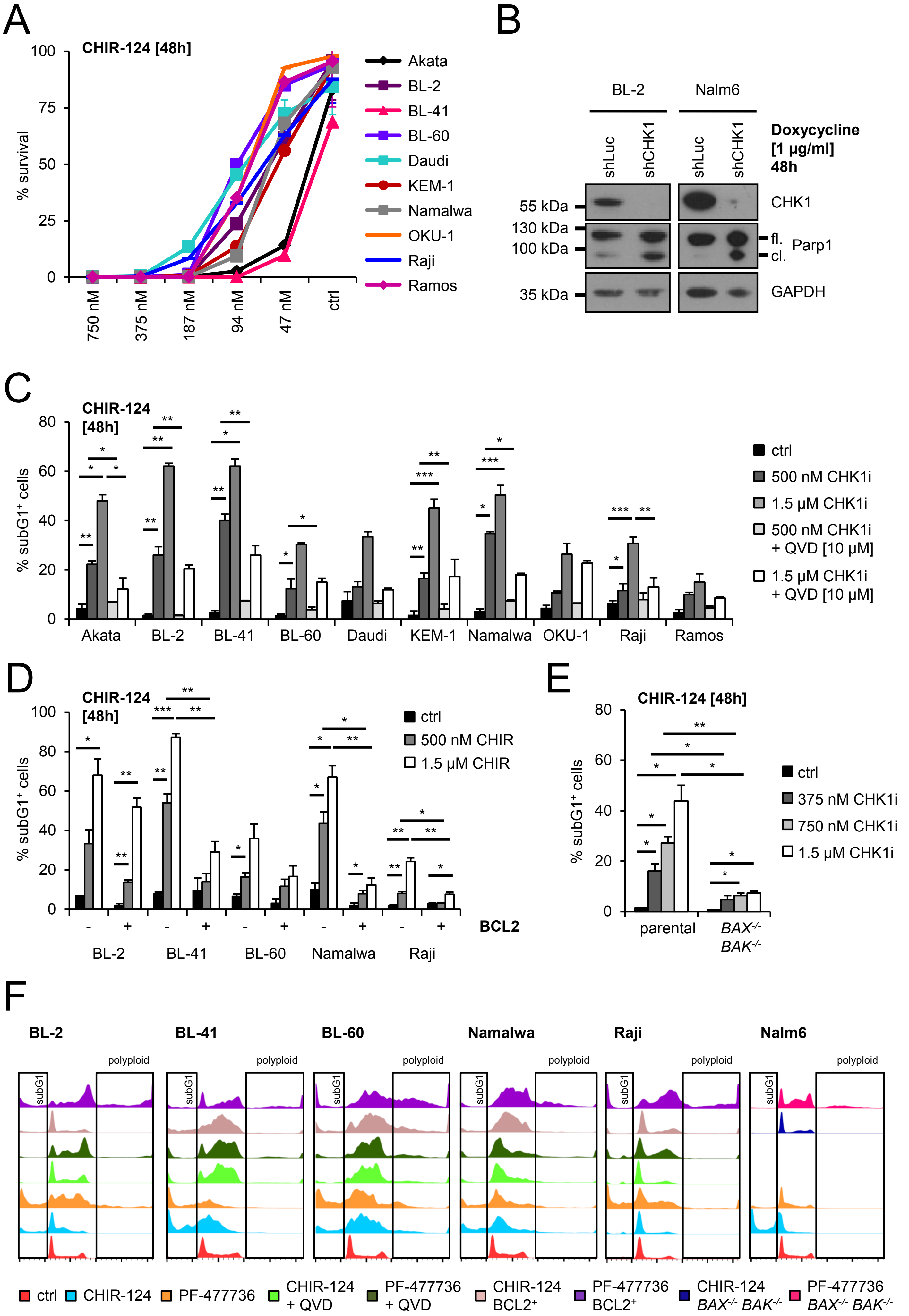
A
CHIR-124 [48h]
% survival
750 nM
375 nM
187 nM
94 nM
47 nM
ctrl
Akata
BL-2
BL-41
BL-60
Daudi
KEM-1
Namalwa
OKU-1
Raji
Ramos
B
BL-2
Nalm6
shLuc
shCHK1
Doxycycline [1 µg/ml] 48h
55 kDa
130 kDa
100 kDa
35 kDa
CHK1
fl.
cl.
Parp1
GAPDH
C
CHIR-124 [48h]
% subG1⁺ cells
ctrl
500 nM CHK1i
1.5 µM CHK1i
500 nM CHK1i + QVD [10 µM]
1.5 µM CHK1i + QVD [10 µM]
D
CHIR-124 [48h]
500 nM CHIR
1.5 µM CHIR
BCL2
E
375 nM CHK1i
750 nM CHK1i
parental
BAX-/- BAK-/-
F
Nalm6
polyploid
subG1
CHIR-124 + QVD
PF-477736 + QVD
CHIR-124 BCL2⁺
PF-477736 BCL2⁺
CHIR-124 BAX-/- BAK-/-
PF-477736 BAX-/- BAK-/-

Supplementary Figure 1: Inhibition of CHK1 results in BCL2-regulated apoptotic cell death (A) Dose response of Burkitt lymphoma cells lines to CHK1 inhibitor CHIR-124. Cell survival was assessed using AnnexinV-staining and flow cytometry. (B) Burkitt lymphoma cell line BL-2 and pre-B ALL cell line Nalm6 (both p53-proficient) were lentivirally transduced with an shRNA against CHK1 (shCHK1) or Luciferase (shLuc) and selected on puromycin resistance. Cells were then treated for 48 h with 1 µg/ml doxycycline to induce shRNA expression and then processed for western analysis using antibodies specific for the indicated genes. (C) Burkitt lymphoma cells lines were treated for 48h with the CHK1 inhibitor CHIR-124, either alone or in combination with the pan-caspase inhibitor QVD. Cells were processed for sub-G1 analysis using PI staining and flow cytometry. (D) Cell death analysis using BCL2 overexpressing or control virus-transduced Burkitt lymphoma cell lines. Sub-G1 levels were assessed 48h after treatment with the CHK1 inhibitor CHIR-124 or solvent control (DMSO). (E) Nalm6 pre-B ALL cells deficient for BAX and BAK were treated with CHK1 inhibitor CHIR-124 or solvent control (DMSO) and sub-G1 fractions were compared to parental cells. (F) Representative DNA-profiles of Burkitt lymphoma cell lines ± exogenous BCL2 and Nalm6 pre-B ALL cells ± BAX/BAK. Burkitt cell lines were treated with 500 nM of CHIR-124 or PF-477736; Nalm6 cells were treated with 1.5 µM of inhibitor. QVD was used at a final concentration of 10 µM. Bars represent means ± S.E.M (n=3/treatment and genotype), * equals $p < 0.05$, ** $p < 0.01$, *** $p < 0.001$ using unpaired Student´s t-test.

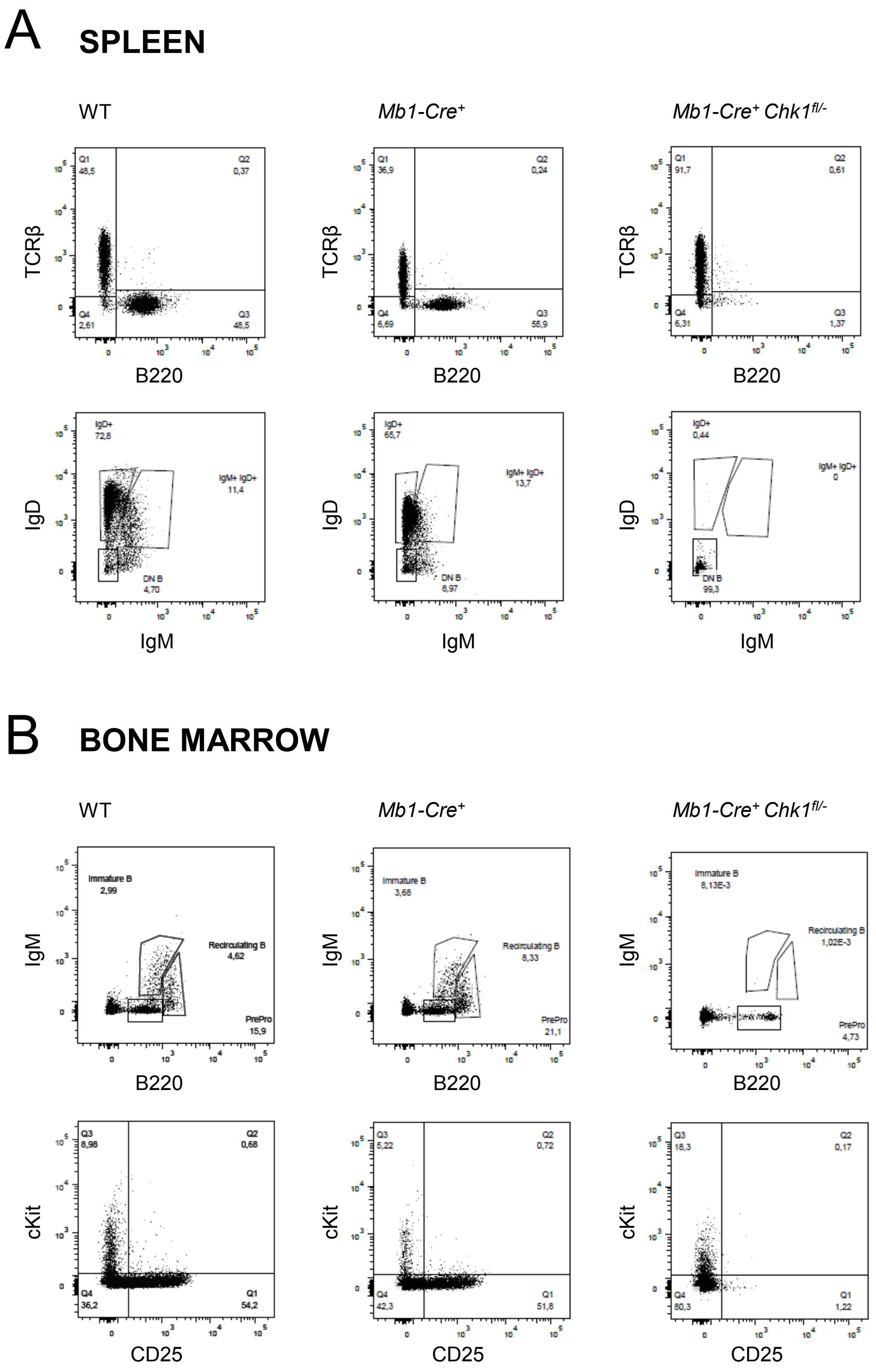
A
SPLEEN
WT
Mb1-Cre+
Mb1-Cre+ Chk1fl/-
TCRβ
B220
Q1 48,5
Q2 0,37
Q3 48,5
Q4 2,61
Q1 36,9
Q2 0,24
Q3 55,9
Q4 6,69
Q1 91,7
Q2 0,61
Q3 1,37
Q4 6,31
IgD
IgM
IgD+ 72,8
IgM+ IgD+ 11,4
DN B 4,70
IgD+ 65,7
IgM+ IgD+ 13,7
DN B 8,97
IgD+ 0,44
IgM+ IgD+ 0
DN B 99,3
B
BONE MARROW
WT
Mb1-Cre+
Mb1-Cre+ Chk1fl/-
IgM
B220
Immature B 2,99
Recirculating B 4,62
PrePro 15,9
Immature B 3,68
Recirculating B 8,33
PrePro 21,1
Immature B 8,13E-3
Recirculating B 1,02E-3
PrePro 4,73
cKit
CD25
Q3 8,98
Q2 0,68
Q4 36,2
Q1 54,2
Q3 5,22
Q2 0,72
Q4 42,3
Q1 51,8
Q3 18,3
Q2 0,17
Q4 80,3
Q1 1,22

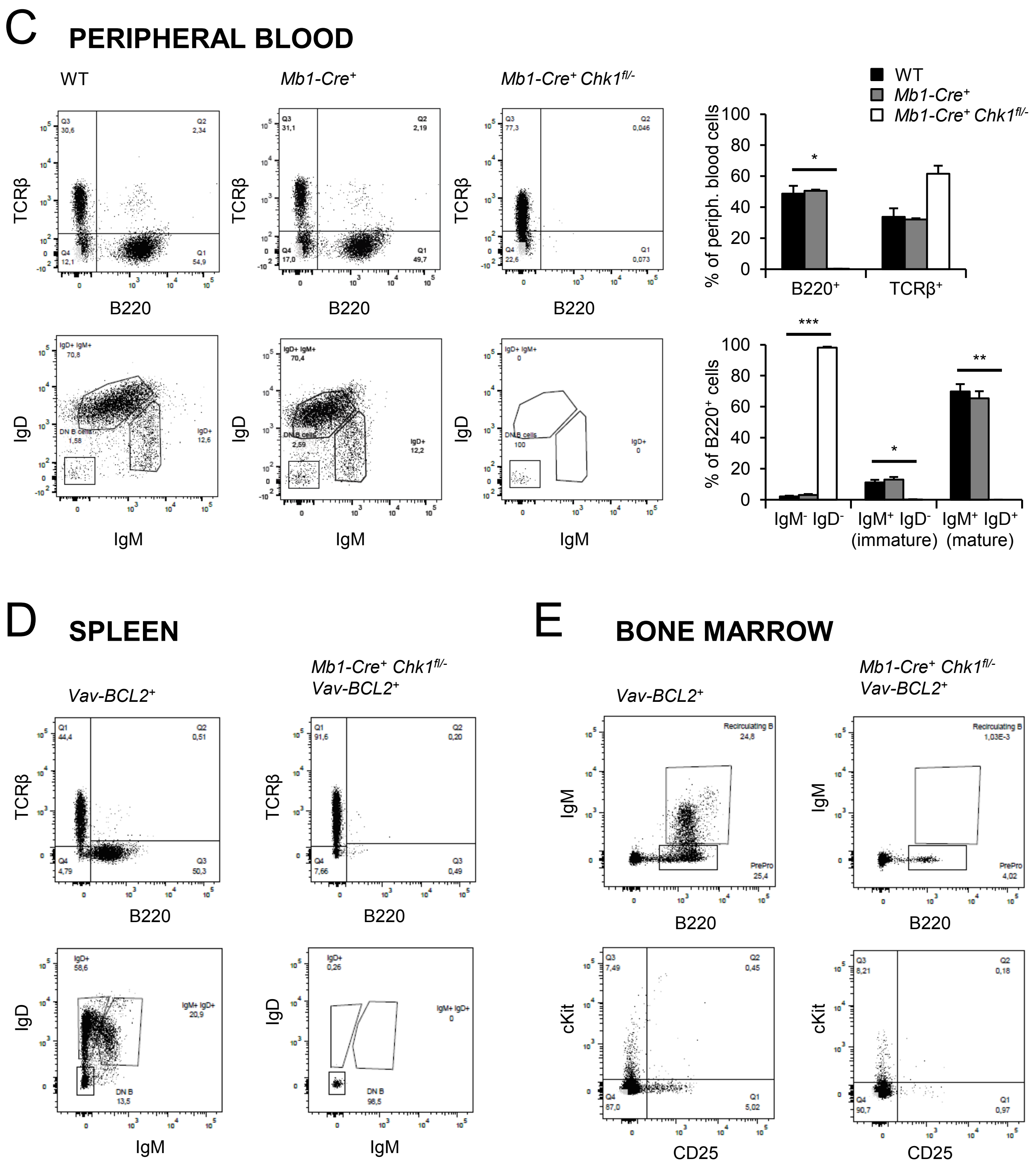

Supplementary Figure 2: Chk1 is essential for early B cell development Representative dot plots of (A) spleens and (B) bone marrow derived from mice of the indicated genotypes. (A) anti-B220 and anti-TCRβ-specific antibodies were used to determine the relative percentages of B cells and T cells, respectively, anti-IgM and anti-IgD antibodies were used discriminate different developmental stages within the $B220^+$ B cell pool. (B) Anti-B220 and anti-IgM Abs were used to determine bone marrow pro/pre-B cells ($B220^+$ IgM^-) that can be further characterized by their expression of cKit and CD25 in $B220^+$ IgM^- $cKit^+$ $CD25^-$ pro B and $B220^+$ IgM^- $cKit^-$ $CD25^+$ pre-B cells. (C) Representative dot plots and quantification of peripheral blood cells analyzed as in (B). (D) Representative dot plots of BCL2 transgenic splenocytes proficient or deficient for CHK1 in B cells. (E) Representative dot plots of bone marrow cells of the indicated genotypes. Bars represent means of n=3 animals ± S.E.M. *equals $p < 0.05$, ** $p < 0.01$, *** $p < 0.001$ using unpaired Student´s t-test.

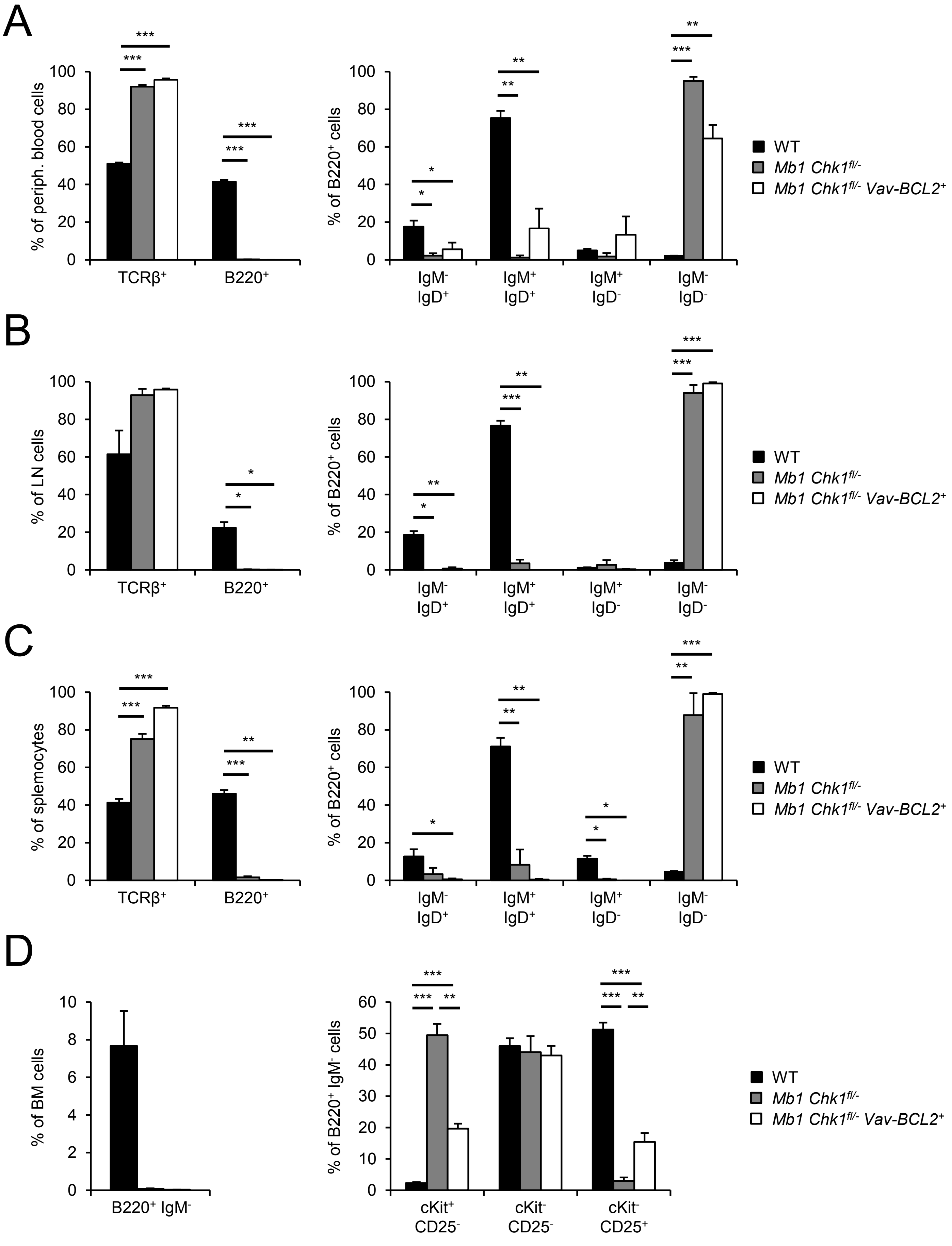

Supplementary Figure 3: Loss of Chk1 imposes a permanent B cell differentiation block. (A) Peripheral blood, (B) lymph nodes (LN), (C) spleen and (D) bone marrow of aged mice (8-12 months) were analyzed for B cell subset composition by flow cytometry using the indicated antibodies. (A-D) WT n=3, *Mb1-Cre*$^{+}$ *Chk1*$^{fl/-}$ n=4, *Mb1-Cre*$^{+}$ *Chk1*$^{fl/-}$ *Vav-BCL2*$^{+}$ n=5 ± S.E.M. * equals $p< 0.05$, ** $p < 0.01$, *** $p < 0.001$ using unpaired Student´s t-test.

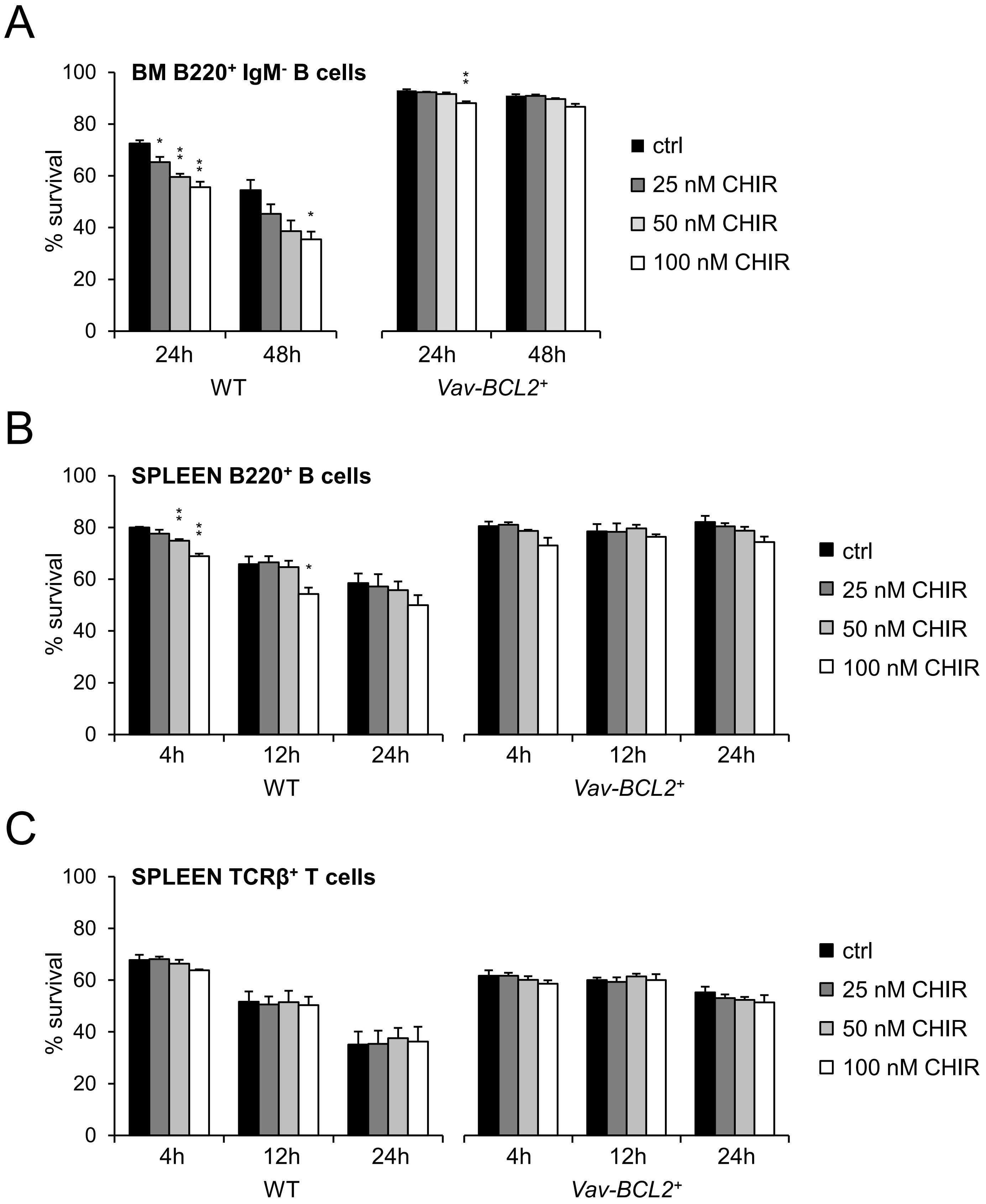

Supplementary Figure 4: Overexpression of anti-apoptotic BCL2 blocks CHK1 inhibition-mediated cell death ex vivo (A) FACS sorted wild-type (WT) and BCL2 transgenic (*Vav-BCL2$^+$*) bone marrow derived B220$^+$ IgM$^-$ pro/pre B cells were treated for 24h and 48h with CHIR-124 (CHK1i) or solvent control (DMSO). Cells were processed for survival analysis using a flow cytometer and AnnexinV/7AAD staining. (B,C) Total splenocytes were cultured for the indicated time in the presence of CHIR-124 (CHK1i) or solvent control (DMSO) and processed for survival analysis using B220/TCRβ/AnnexinV-staining and flow cytometry. (B) Quantification of B220$^+$ AnnexinV$^-$ cells. (C) Quantification of TCRβ$^+$ AnnexinV$^-$ cells. Bars represent means of n=3 animals/genotype ± S.E.M. * equals $p < 0.05$, ** $p < 0.01$, *** $p < 0.001$ using unpaired Student´s t-test.

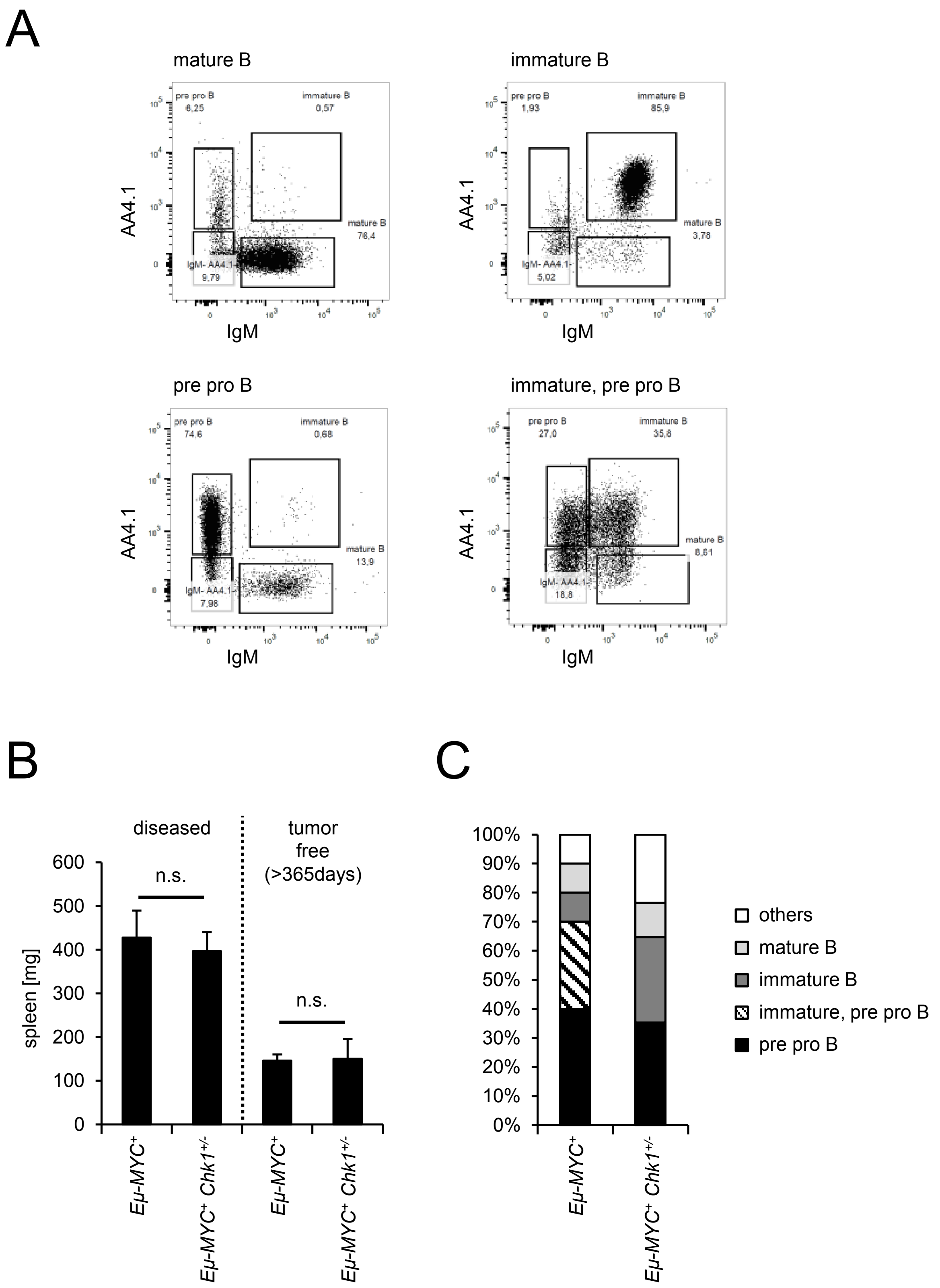

Supplementary Figure 5: Successful lymphocyte transformation depends on CHK1 expression levels. (A) Representative dot plots of different tumor immune phenotypes found in Eμ-MYC transgenic mice: mature B cell lymphomas (CD19$^+$ AA4.1$^-$ IgM$^+$), immature IgM$^+$ B cell lymphomas (CD19$^+$ AA4.1$^+$ IgM$^+$), pro/pre B (CD19$^+$ AA4.1$^+$ IgM$^-$) and mixed lymphomas. (B) Quantification of spleen weights found in diseased (palpable tumors) *Eμ-MYC$^+$* (n=8), *Eμ-MYC$^+$ Chk1$^{+/-}$* (n=10) or 1-year-old tumor-free mice *Eμ-MYC$^+$* (n=3), *Eμ-MYC$^+$ Chk1$^{+/-}$* (n=5). Bars represent means ± S.E.M. (C) Quantification of immune phenotypes of diseased mice of the indicated genotypes (*Eμ-MYC$^+$* n=10, *Eμ-MYC$^+$ Chk1$^{+/-}$* n=17). n.s. = not significant.

A

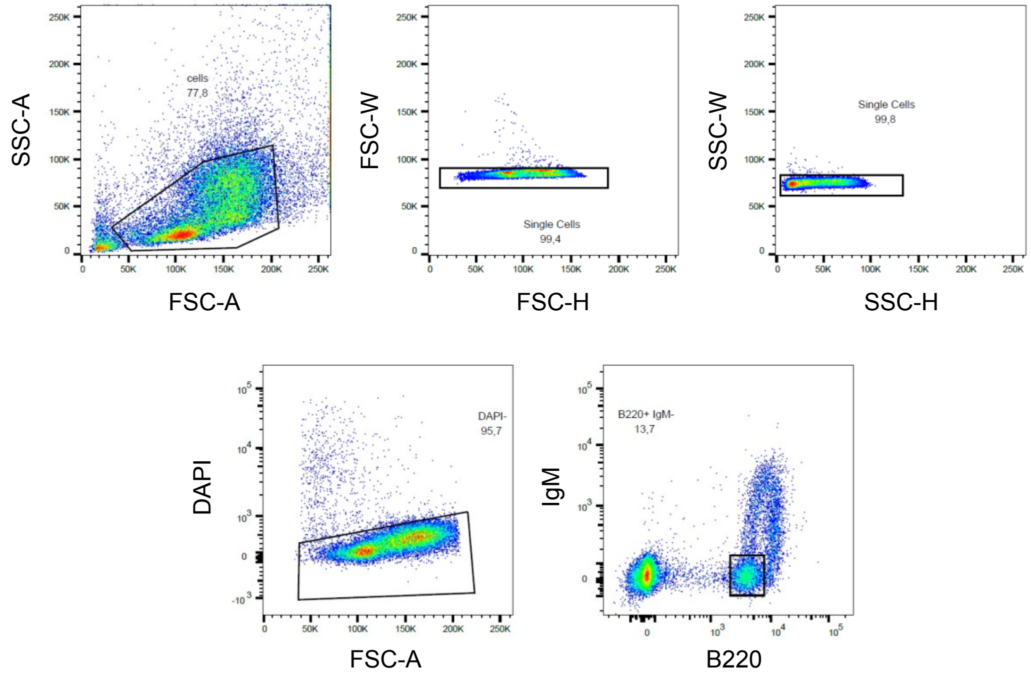

B

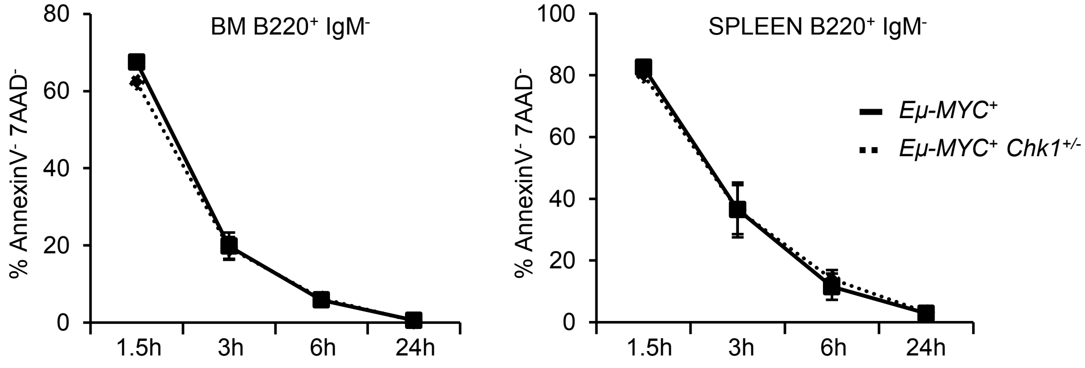

Supplementary Figure 6: Analysis of spontaneous cell death of sorted B cells from premalignant mice. (A) Gating strategy used for sorting bone marrow-derived B220$^+$ IgM$^-$ pro/pre B cells. (B) FACS sorted bone marrow and splenic B220$^+$ IgM$^-$ pro/pre B cells of pre-malignant *Eμ-MYC$^+$* and *Eμ-MYC$^+$ Chk1$^{+/-}$* mice were processed for an ex vivo survival assay and analyzed using a flow cytometer and AnnexinV/7AAD staining (n=3).

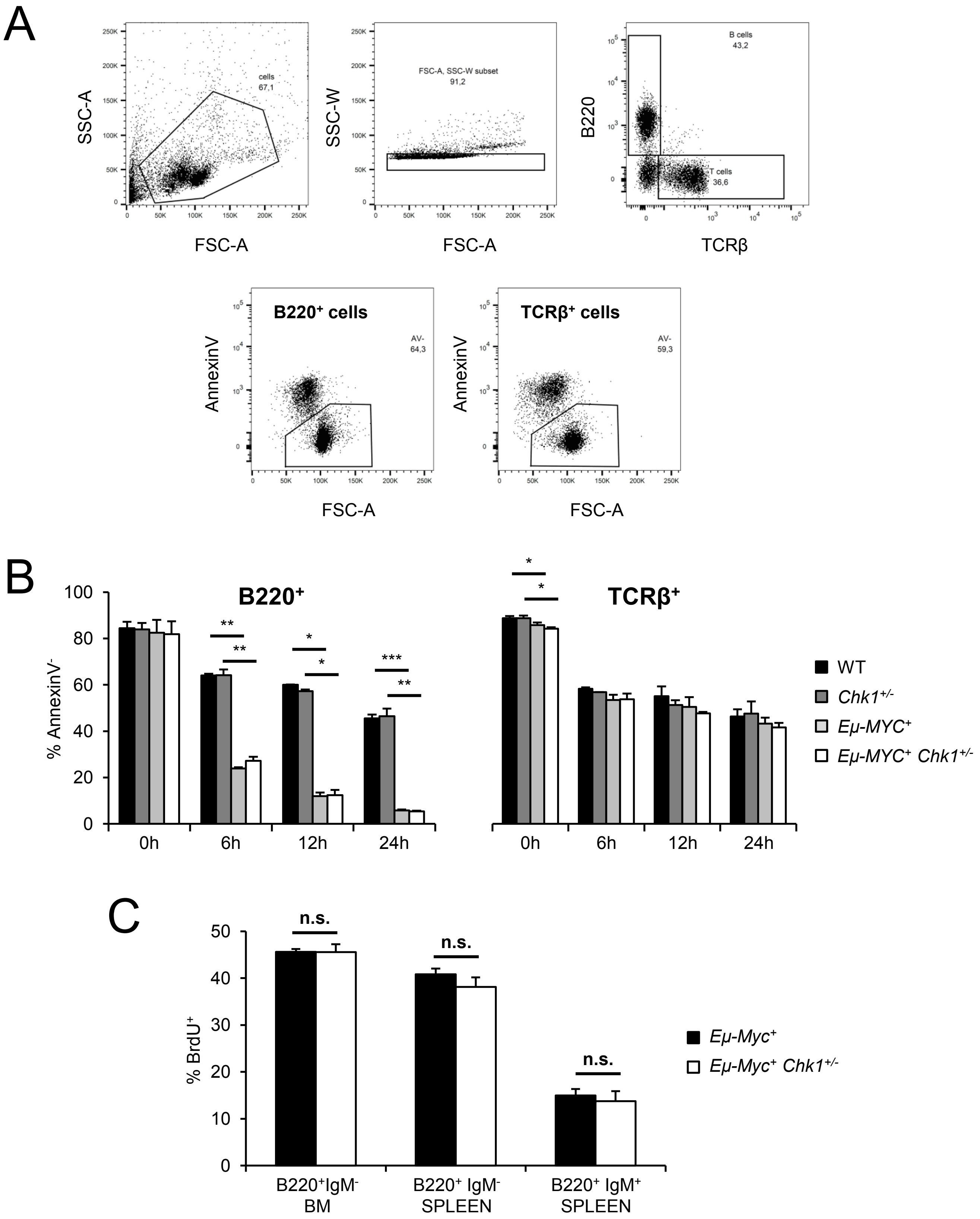

Supplementary Figure 7: Analysis of splenocyte apoptosis *ex vivo* and BrdU-incorporation rates in premalignant mice (A) Layout used to discriminate living (Annexin V^-) B cells ($B220^+$) and T cells ($TCR\beta^+$) in the spleen as quantified in Fig. 3 and S3. (B) Cell death of total splenocytes of indicated genotypes (n=4) was assessed using flow cytometry and B220/TCRβ/AnnexinV staining. (C) Proliferation of different B cell subsets in bone marrow or spleen of the indicated genotypes *in vivo* was assessed four hours after intraperitoneal BrdU injection. $BrdU^+$ cells were analyzed via intracellular FACS staining. n=3/4 (*Eμ-MYC*$^+$/*Eμ-MYC*$^+$ *Chk1*$^{+/-}$) ± S.E.M. n.s. = not significant, * equals $p < 0.05$, ** $p < 0.01$, *** $p < 0.001$ using unpaired Student´s t-test.

<u>Fig 1 A</u>

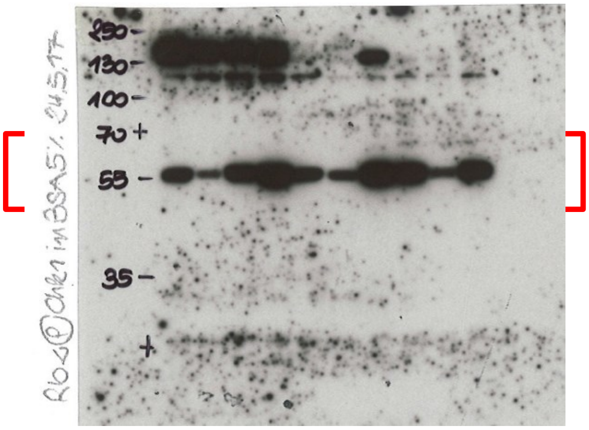

pChk1 Ser345

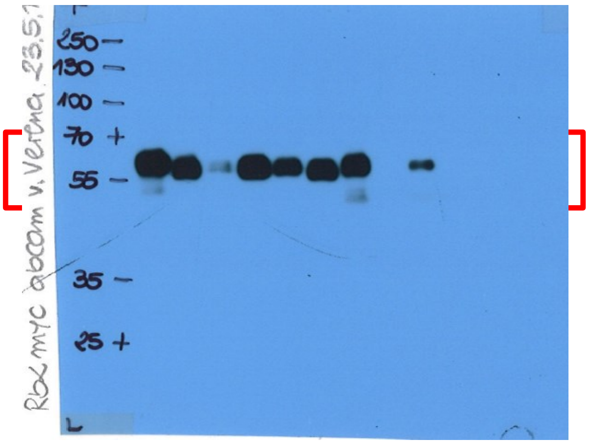

MYC

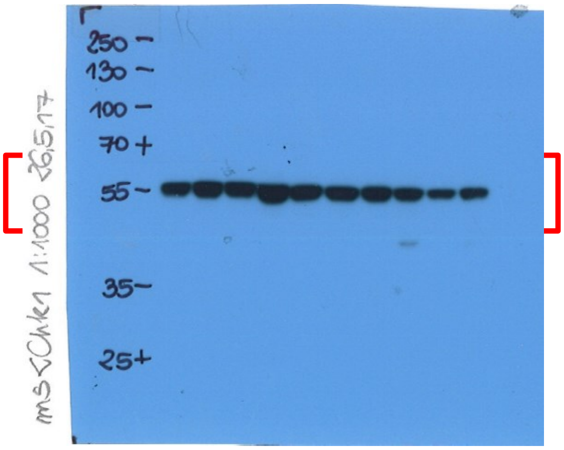

CHK1

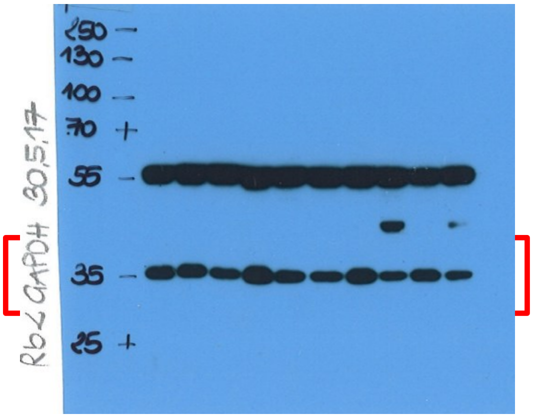

GAPDH

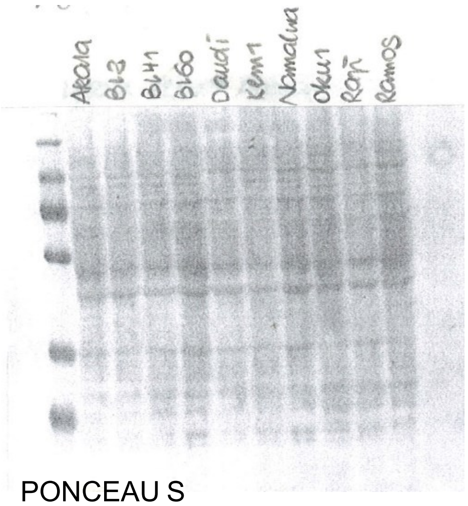

PONCEAU S

Supplementary Figure 8: Uncropped scans of most important blots displayed in various main figures

Fig 2 B

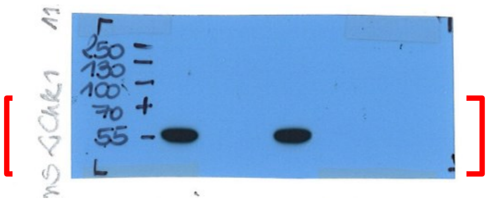

CHK1

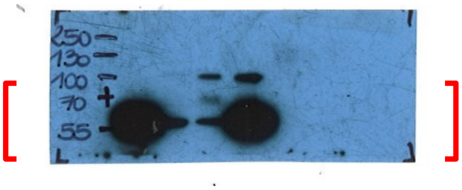

CHK1

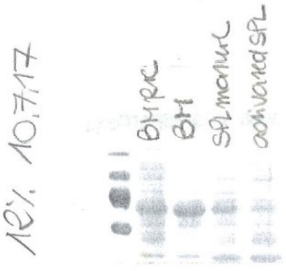

PONCEAU S

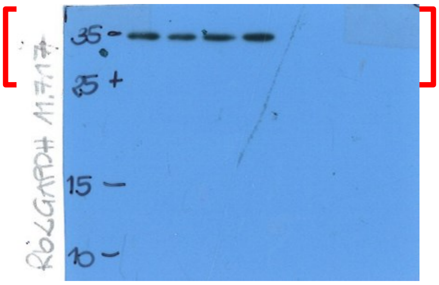

GAPDH

Fig 5 A experiment 2

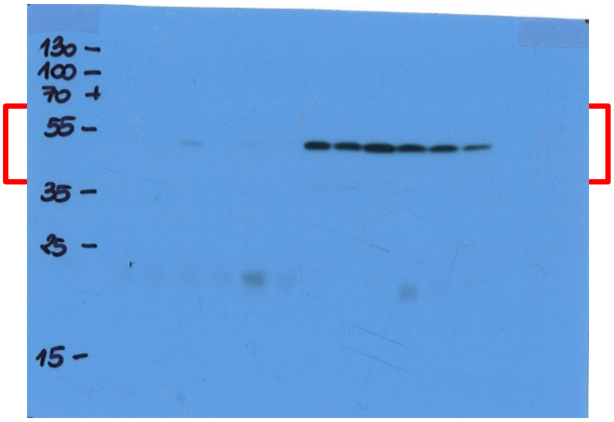

CHK1 (* also used in FIG 6A)

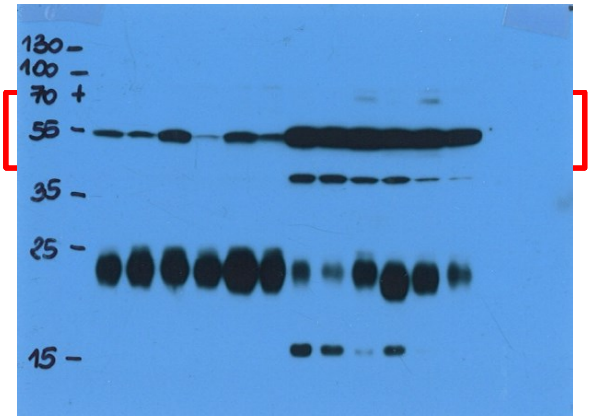

CHK1 (longer exposure)

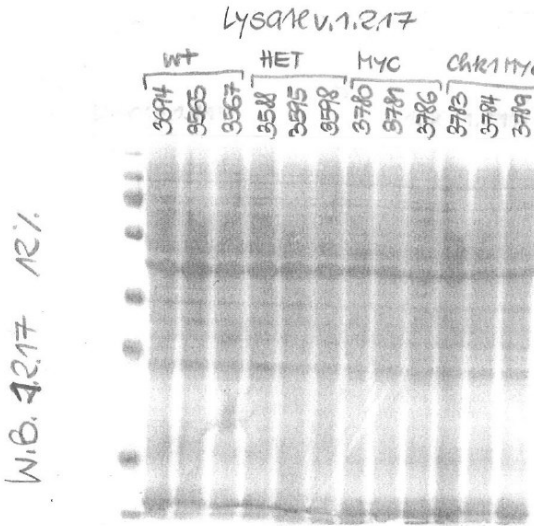

PONCEAU S

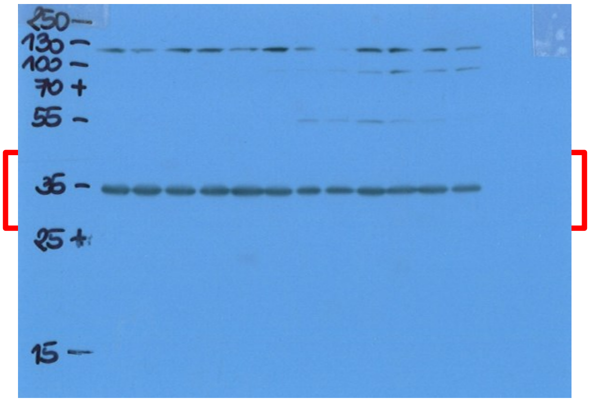

GAPDH

Supplementary Figure 8: Uncropped scans of most important blots displayed in various main figures

Fig 5 C Spleen

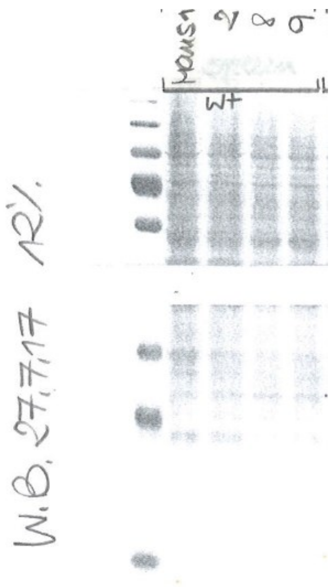

PONCEAU S

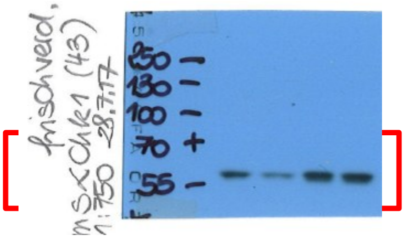

CHK1

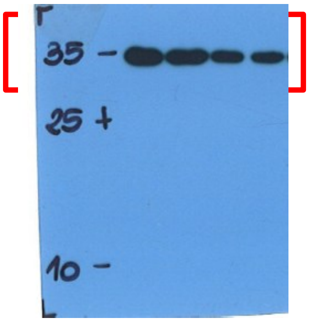

GAPDH

Fig 5 C Thymus

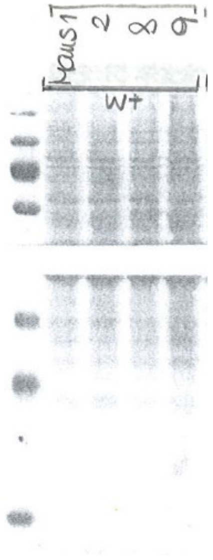

PONCEAU S

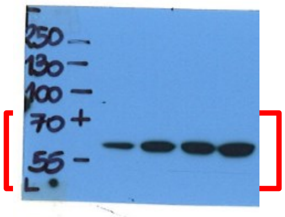

CHK1

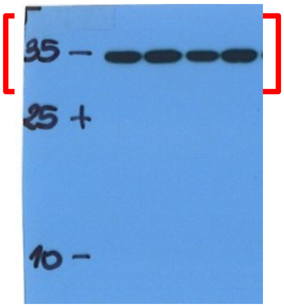

GAPDH

Supplementary Figure 8: Uncropped scans of most important blots displayed in various main figures

Fig 6 A: left (lane 1-4!)

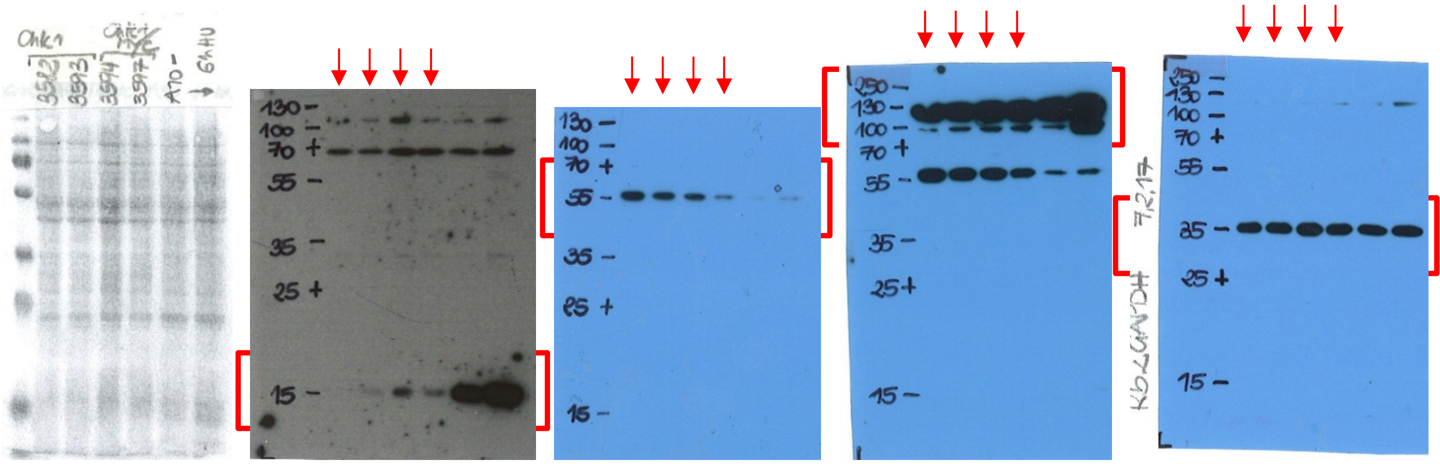

PONCEAU S | gH2A.X | CHK1 | Parp1 | GAPDH

Fig 6 A: right (lane 7-12!)

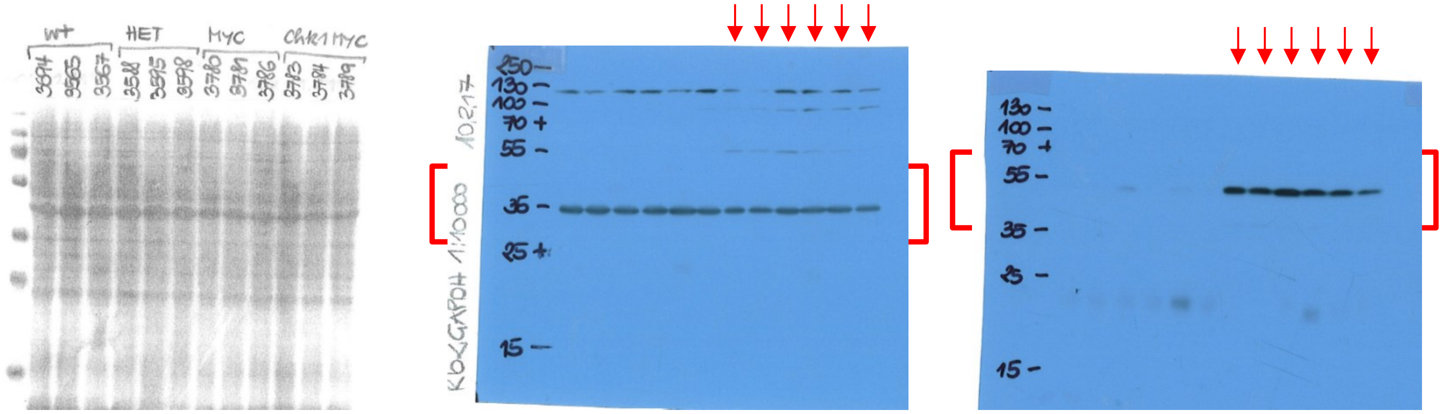

PONCEAU S | GAPDH | CHK1 (* also used in FIG 5A)

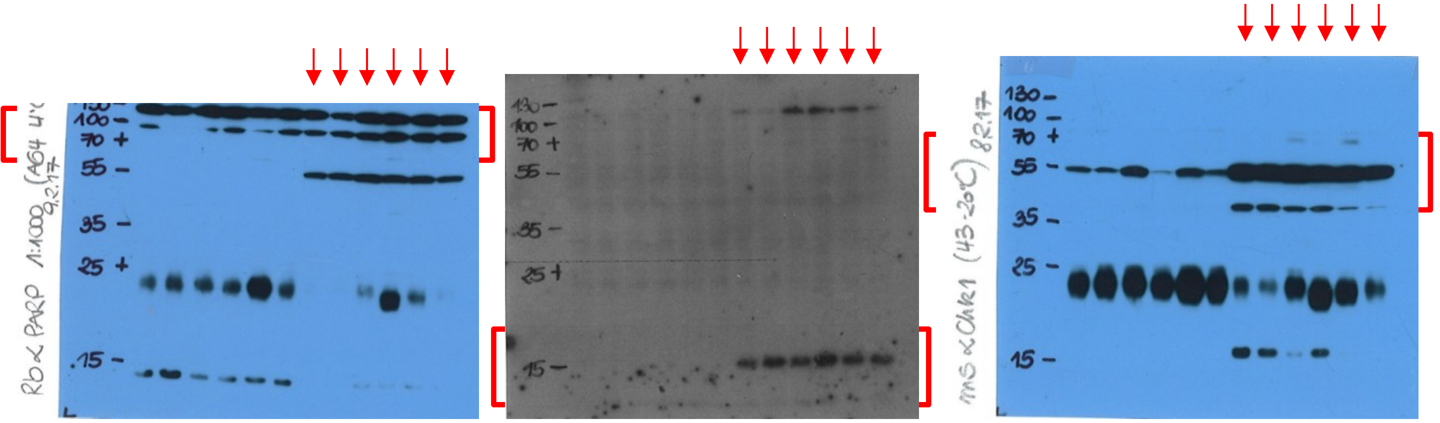

Parp1 | gH2AX | CHK1 (longer exposure)

Supplementary Figure 8: Uncropped scans of most important blots displayed in various main figures

Fig 7 A (lane 1-2!)

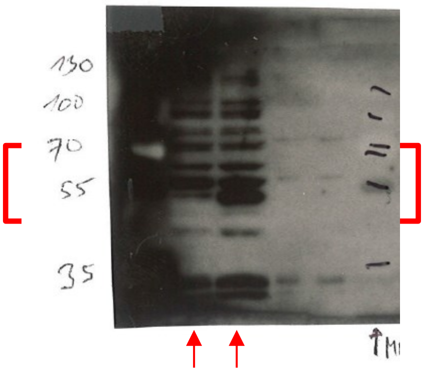

p53

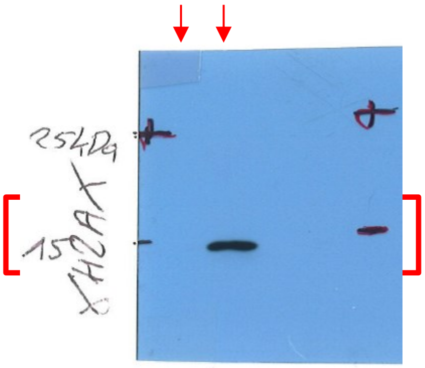

gH2AX

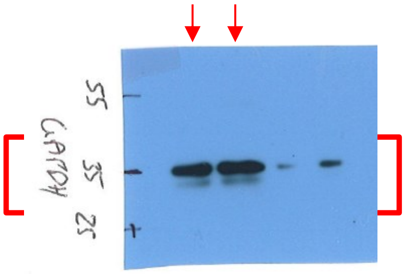

GAPDH

Fig 7 B

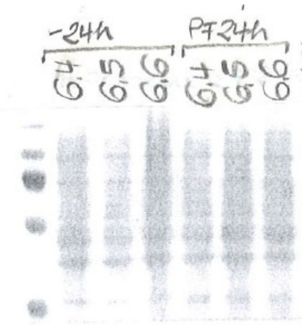

PONCEAU S

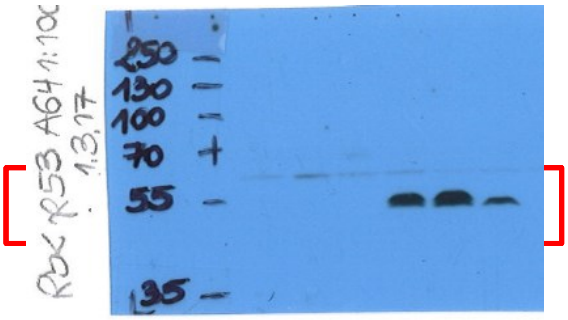

p53

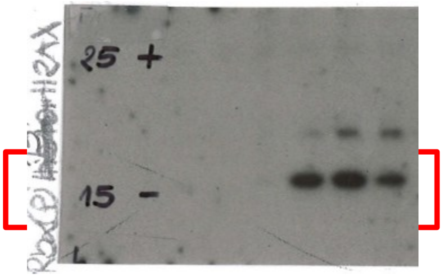

gH2AX

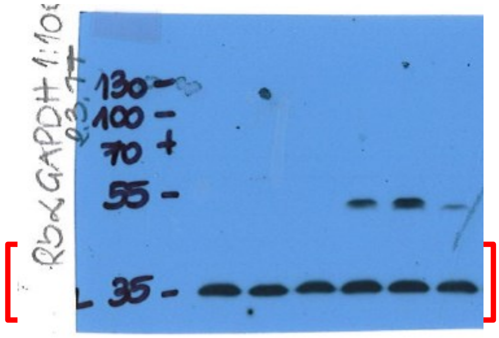

GAPDH

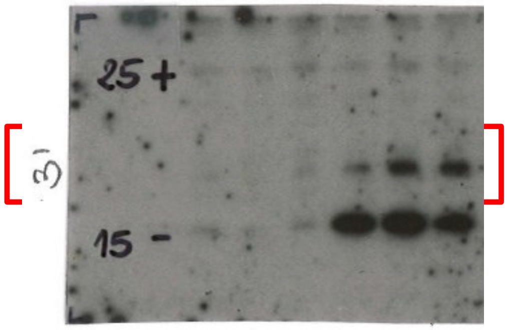

p21

Fig 7 C (lane 2+3!)

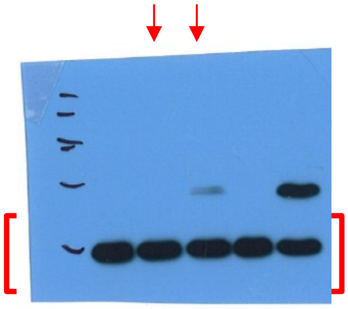

GAPDH

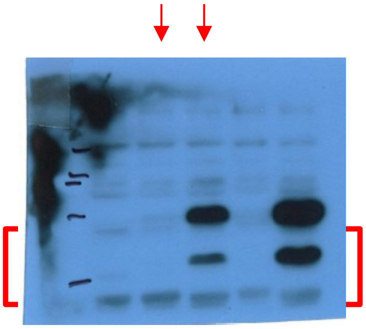

gH2AX

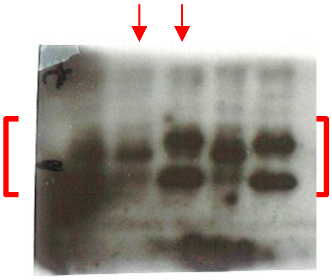

p21

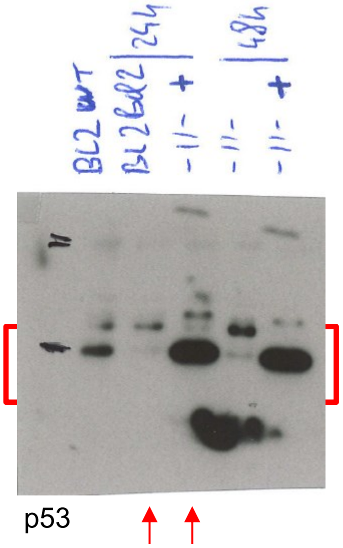

p53

Supplementary Figure 8: Uncropped scans of most important blots displayed in various main figures

SUPPL FIG 1 B (lane 1, 2, 5 and 6!)

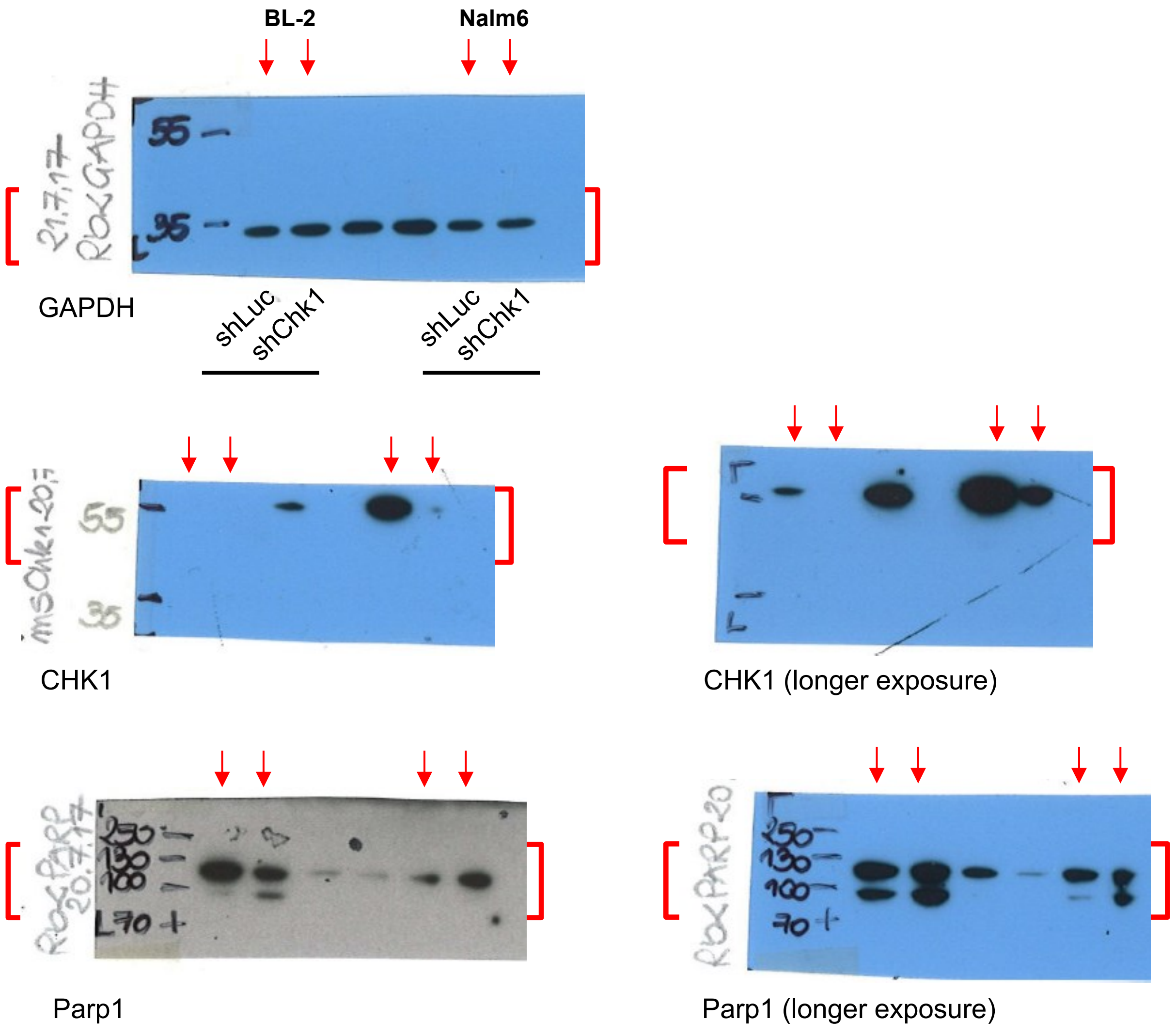

Supplementary Figure 8: Uncropped scans of most important blots displayed in various main figures
